# LINC00520: A Potential Diagnostic and Prognostic Biomarker in Cancer

**DOI:** 10.3389/fimmu.2022.845418

**Published:** 2022-03-02

**Authors:** Qiudan Zhang, Jinze Shen, Yuchen Wu, Wenjing Ruan, Feng Zhu, Shiwei Duan

**Affiliations:** ^1^ School of Medicine, Zhejiang University City College, Hangzhou, China; ^2^ Medical Genetics Center, School of Medicine, Ningbo University, Ningbo, China; ^3^ Department of Clinical Medicine, The First School of Medicine, Wenzhou Medical University, Wenzhou, China; ^4^ Department of Respiratory Diseases, Sir Run Run Shaw Hospital, School of Medicine, Zhejiang University, Hangzhou, China

**Keywords:** LINC00520, ceRNA, transcription factor, P13K/AKT, JAK/STAT, prognosis

## Abstract

Long non-coding RNA (lncRNA) is important in the study of cancer mechanisms. LINC00520 is located on human chromosome 14q22.3 and is a highly conserved long non-coding RNA. LINC00520 is widely expressed in various tissues. The expression of LINC00520 is regulated by transcription factors such as Sp1, TFAP4, and STAT3. The high expression of LINC00520 is significantly related to the risk of 11 cancers. LINC00520 can competitively bind 10 miRNAs to promote tumor cell proliferation, invasion, and migration. In addition, LINC00520 is involved in the regulation of P13K/AKT and JAK/STAT signaling pathways. The expression of LINC00520 is significantly related to the clinicopathological characteristics and prognosis of tumor patients and is also related to the sensitivity of HNSCC to radiotherapy. Here, this article summarizes the abnormal expression pattern of LINC00520 in cancer and its potential molecular regulation mechanism and points out that LINC00520 can be used as a potential biomarker for cancer diagnosis, prognosis, and treatment.

## Introduction

Long non-coding RNA (lncRNA) is a transcript with a length of more than 200 nucleotides. LncRNA regulates gene expression at the transcriptional, post-transcriptional, and epigenetic levels (1, 2), which is important for elucidating the molecular mechanism of cancer (3).

The long non-coding RNA 520 (LINC00520) is located on human chromosome 14q22.3. LINC00520 is a highly conserved lncRNA that is widely expressed in various tissues (4). LINC00520 has great potential in cancer diagnosis, prognostic evaluation, and treatment. The current study found that LINC00520 is abnormally expressed in a variety of cancers. Besides, LINC00520 is significantly highly expressed in acute kidney injury (AKI) (5).

lncRNA can be used as competitive endogenous RNA (ceRNA) to interfere with miRNA-induced gene silencing (6). As the ceRNA of 10 miRNAs, LINC00520 can regulate the proliferation, invasion, and apoptosis of a variety of cancer cells. Like many protein-coding genes, expression of lncRNA is also regulated by transcription factors (7). The expression of LIN00520 is regulated by transcription factors such as SP1, TFAP4, and STAT3 (8–10).

More and more studies have shown that LINC00520 plays an important role in the occurrence and development of tumors. Here, this article provides a detailed review of the research progress, molecular mechanism, and clinical significance of LINC00520, aiming to clarify the important role of LINC00520 in tumor diagnosis and prognosis, and provide clues for the future research of LINC00520.

## The Aberrant Expression of LINC00520 in Cancer

LINC00520 is abnormally upregulated in 11 cancers, including lung cancer (11), non-small cell lung cancer (NSCLC) (10), lung adenocarcinoma (LUAD) (12, 13), breast cancer (BC) (4, 8), papillary thyroid carcinoma (PTC) (14), colorectal cancer (CRC) (15), melanoma (MM) (16–18), head and neck squamous cell carcinoma (HNSCC) (19), laryngeal squamous cell carcinoma (LSCC) (20), glioma (9), nasopharyngeal carcinoma (NPC) (21), etc. Further *in vitro* experiments have shown that LINC00520 significantly promotes tumor progression by controlling the proliferation and other biological functions of cancer cells (14, 21) (**Table 1**).

**Table 1:** The role of LINC00520 in various cancers.

Tumor type	Number of clinical samples	Assessed cell line	The expression of LINC00520	Effect *in vivo*	Effect *in vitro*	Regulatory mechanism	Ref.
Lung cancer	52 paired tissues	H460, H1975, H1299, A549, and BEAS-2B	Up-regulation	—	migration↑, invasion↑, proliferation↑, cell cycle↑, apoptosis↓	LINC00520/miR-3175	(11)
BC		MCF10A, SKBR3, MCF7, MDA-MB-468, MDA-MB-453, MDA-MB-231, T47D, BT549, and SUM159-PT	Up-regulation	—	migration↑, invasion↑	STAT3 promote LINC00520	(8)
						PI3K signaling pathway promote LINC00520	
	504 cases with BC and 505 healthy controls	293T	Up-regulation	—		LINC00520/miR-3122	(4)
PTC	59 paired tissues	HT-ori3, HTH83, K-1, BCPAP, and TPC-1	Up-regulation	tumor growth ↑	migration↑, invasion↑, apoptosis↓	LINC00520/miR-577/Sphk2	(14)
CRC	132 paired tissues	NCM460, HCT116, SW480, HT29, and Lovo	Up-regulation	—	colony forming ability↑, migration↑, invasion↑, proliferation↑, apoptosis↓	LINC00520/miR-577/HSP27	(15)
cSCC		A431	De-regulation	tumor growth ↓	migration↓, invasion↓, proliferation↓, adhesion↓	LINC00520/EGFR/PI3K/AKT	(22)
MM			Up-regulation	—			(18)
			Up-regulation		EMT↑	LINC00520/let-7a-5p/DTL	(17)
	41 paired tissues	A375, A2058, MeWo, CHL-1, and SK-MEL-28	Up-regulation	tumor growth ↑ pro-metastasis	migration↑, invasion↑, proliferation↑, EMT↑	LINC00520/miR-125b-5p/EIF5A2	(16)
				activity ↑			
LUAD	20 paired tissues	A549, H1975, H2030, H1435, and BEAS-2B	Up-regulation	—	migration↑, invasion↑	LINC00520/miR-3611/FOXP3	(13)
	30 paired tissues	A549, H358, H1299, and HBE	Up-regulation	tumor growth ↑	migration↑, invasion↑, proliferation↑	LINC00520/miR-1252-5p/FOXR2	(12)
HNSCC	40 paired tissues	SCC-25, PCI-37B, TU686, and TE-10	Up-regulation	tumor growth ↑	migration↑, invasion↑, proliferation↑, apoptosis↓	LINC00520/miR-195/HOXA10	(19)
LSCC	65 paired tissues		Up-regulation	—			(20)
glioma	12 cases with grade I‐II, 12 cases with grade III‐IV, and 12 health controls	U87, U251, and HEK293	Up-regulation	tumor growth ↑	migration↑, invasion↑, proliferation↑, apoptosis↓	TFAP4/LINC00520/miR-520f-3p	(9)
NPC	50 paired tissues	SUNE-1, CNE-1, HNE-1, CNE-2, C666-1, HONE-1, and NP69	Up-regulation	tumor growth ↑	proliferation↑	LINC00520/miR-26b-3p/USP39	(21)
NSCLC	150 paired tissues	H1975, H1299, PC 9, HCC827, A549, and BEAS-2B	Up-regulation	tumor growth ↑	migration↑, invasion↑, proliferation↑, apoptosis↓	LINC00520/miR-577/CCNE2	(10)
					EMT↑		

BC, breast cancer; PTC, papillary thyroid carcinoma; CRC, colorectal cancer; cSCC, cutaneous squamous cell carcinoma; MM, melanoma; LUAD, lung adenocarcinoma; HNSCC, head and neck squamous cell carcinoma; LSCC, laryngeal squamous cell carcinoma; NPC, nasopharyngeal carcinoma; NSCLC, non-small cell lung cancer; EMT, epithelial-mesenchymal transition.

In contrast to the above, LINC00520 is down-regulated in cutaneous squamous cell carcinoma (cSCC) (22). LINC00520 can inhibit EGFR, and then attenuate the PI3K/Akt signaling pathway, thereby inhibiting the invasion and metastasis of cSCC (22) (**Table 1**).

## The Association of LINC00520 Expression With the Prognostic Value and Clinic-Pathological Features in Cancer Patients

As shown in **Table 2**, LINC00520 can predict a poor prognosis for a variety of cancers. Generally, cancer patients with lower LINC00520 expression levels have a higher overall survival rate (OS), including lung cancer (11), LUAD (13), NSCLC (10), PTC (14), CRC (15), MM (16, 18), and NPC (21).

**Table 2:** Clinic-pathological features or prognostic value of LINC00520 dysfunction.

Tumor type	The expression of LINC00520	Clinicopathological characteristics/Chemoresistance	Prognostic value	Ref.
Lung cancer	Up-regulation		Prognostic factor of OS	(11)
PTC	Up-regulation	associated with tumor size, lymph node metastasis, and TNM stage	Prognostic factor of OS	(14)
CRC	Up-regulation	associated with tumor size, lymph node metastasis, and TNM stage	Prognostic factor of OS Prognostic factor of DFS	(15)
cSCC	Down-regulation	associated with tumor volumes, weights, and lymph node metastasis		(22)
MM	Up-regulation		Prognostic factor of OS	(18)
	Up-regulation	associated with lymph node metastasis	Prognostic factor of OS	(16)
LUAD	Up-regulation		Prognostic factor of OS	(13)
HNSCC	Up-regulation	associated with radiosensitivity		(19)
LSCC	Up-regulation	associated with lymph node metastasis		(20)

PTC, papillary thyroid carcinoma; CRC, colorectal cancer; cSCC, cutaneous squamous cell carcinoma; MM, melanoma; LUAD, lung adenocarcinoma; HNSCC, head and neck squamous cell carcinoma; LSCC, laryngeal squamous cell carcinoma; OS, overal survivle; DFS, disease-free survival.

Highly expressed LINC00520 is also closely related to the clinicopathological characteristics of cancer patients. In lung cancer, the level of LINC00520 in patients with stage III+IV is higher than that of patients with stage I+II (11). In NSCLC, high LINC00520 levels are significantly associated with advanced tumor stage and positive lymph node metastasis (10). In PTC, the high expression of LINC00520 is significantly related to increased tumor size, lymph node metastasis, and advanced TNM stage of PTC patients (14). In CRC, the high expression of LINC00520 is significantly associated with increased tumor size, lymph node metastasis, and advanced TNM stage (15). In MM, the high expression of LINC00520 is closely related to the advanced clinical stage of MM (16, 18).

The high expression of LINC00520 is also significantly related to the decreased efficacy of radiotherapy. HNSCC is common cancer with a 5-year survival rate of 50%. Radiotherapy has an important impact on the prognosis of HNSCC patients (19). LINC00520 can bind miR-195, thereby up-regulating HOXA10, inhibiting the radiosensitivity of HNSCC, and promoting the proliferation, migration, invasion, and survival of HNSCC cells (19). In xenograft tumor models, the silencing of LINC00520 and the overexpression of miR-195 can inhibit the tumor formation of HNSCC xenografts after radiotherapy (19).

However, in cSCC, LINC00520 overexpression can inhibit tumor growth and lymph node metastasis (22).

## The ceRNA Network of LINC00520 in Cancer

**Figure 1:**
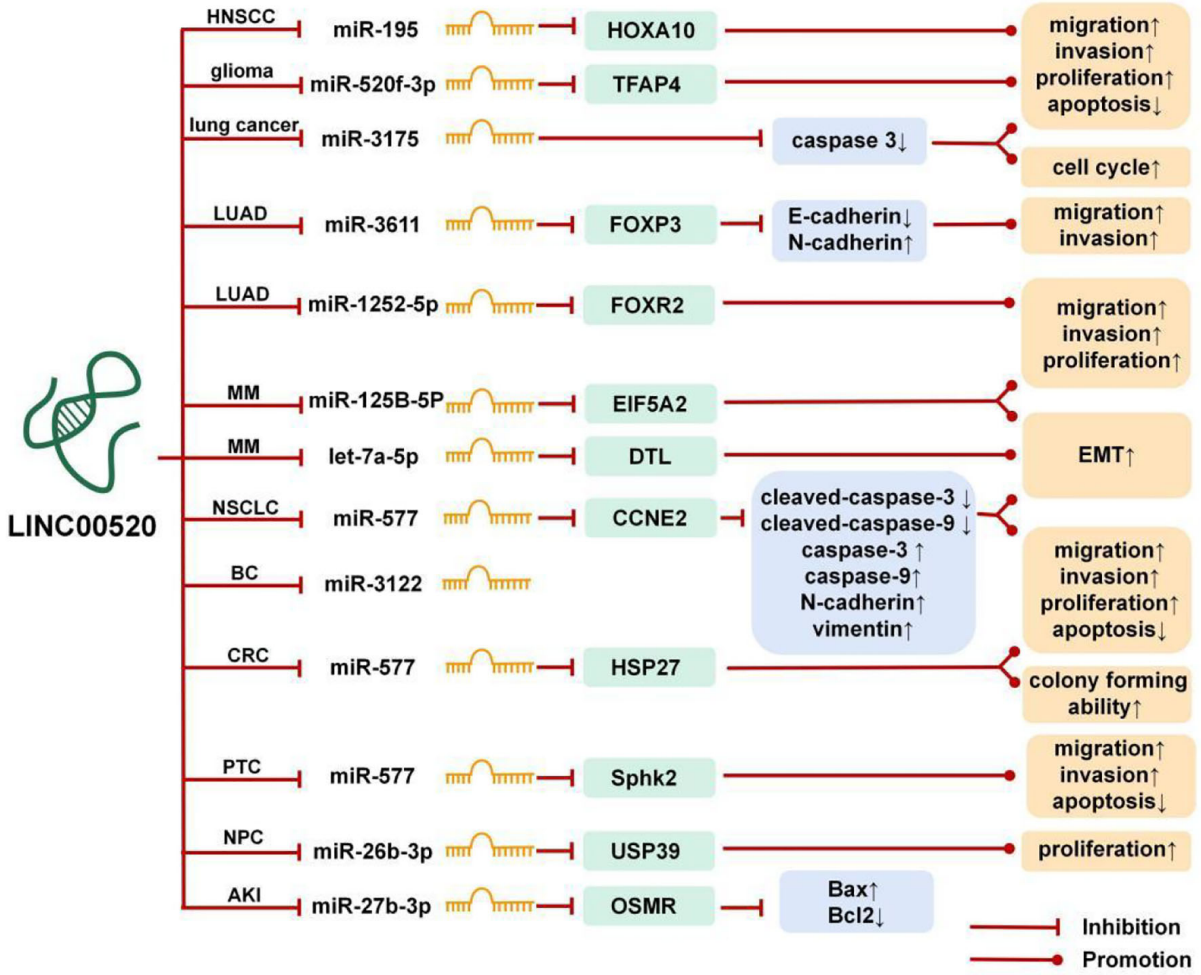
The competing endogenous RNA (ceRNA) mechanism and potential downstream regulatory mechanisms of LINC00520. BC, breast cancer; PTC, papillary thyroid carcinoma; CRC, colorectal cancer; MM, melanoma; LUAD, lung adenocarcinoma; HNSCC, head and neck squamous cell carcinoma; NPC, nasopharyngeal carcinoma; NSCLC, non-small cell lung cancer; AKI, acute kidney injury.

lncRNA can be used as the ceRNA of miRNA, which can affect the expression level of miRNA through the miRNA response elements (MREs) (6). As shown in **Figure 1**, the ceRNA network of LINC00520 involves 10 miRNAs. Through the LINC00520/miRNA/mRNA axis, LINC00520 directly or indirectly regulates the biological behaviors of tumor cells, such as epithelial-mesenchymal transition (EMT), proliferation, invasion, metastasis, apoptosis, etc.

In lung cancer, LINC00520 promotes the proliferation, invasion, and migration of cancer cells by inhibiting the expression of miR-3175 and inhibits cell apoptosis, thereby promoting the occurrence and development of lung cancer (11).

The protein encoded by SPHK2 can catalyze the phosphorylation of sphingosine to sphingosine 1-phosphate, thereby mediating many cellular processes, including migration, proliferation, and apoptosis (23). In PTC, LINC00520 can sponge miR-577 and down-regulation of LINC00520 can increase the expression of miR-577, which leads to a decrease in the expression of SPHK2 and inhibits the occurrence and development of the malignant phenotype of PTC (14).

Heat shock protein 27 (HSP27) is a member of the small heat shock protein family, and overexpression of HSP27 is associated with the promotion of drug resistance, aggressive cancer, metastasis, and poor patient prognosis (24). In CRC, LINC00520 can sponge miR-577, thereby increasing the expression of HSP27 (15).

EIF5A2 can initiate tumor formation, promote cancer cell growth, and increase cancer cell movement and metastasis by inducing EMT (25). In MM, LINC00520 can promote the growth and metastasis of MM *in vivo* through the LINC00520/miR-125b-5p/EIF5A2 axis (16). DTL is important in genome stability and DTL can enhance the proliferation and migration ability of cancer cells (26). In MM, most of the differentially expressed genes were associated with EMT. LINC00520 can promote EMT by regulating the LINC00520/let-7a-5p/DTL axis (17).

FOXP3 and FOXR2 are members of the FOX family. In some cancers, overexpression of FOXP3 significantly induces cell proliferation, migration, and invasion, and its inhibition will impair its carcinogenic function (27). FOXR2 can also promote the migration and invasion of some tumor cells (28). In LUAD, LINC00520 upregulates FOXP3 through sponging miR-3611 to promote LUAD cell proliferation and migration (13). Another study showed that LINC00520 promotes the proliferation, migration, and invasion of LUAD cells by targeting miR-1252-5p to up-regulate FOXR2, thereby promoting the progress of LUAD (12).

HOXA10 is a member of the HOX transcription factor family, which plays an important role in carcinogenesis. The up-regulation of HOXA10 significantly enhances tumor cell proliferation, clone formation, and tumorigenesis, and reduces cell apoptosis (29). In HNSCC, LINC00520 can upregulate the HOXA10 gene by sponging miR-195, thereby reducing the radiosensitivity of HNSCC cells (19). The LINC00520/miR-195/HOXA10 axis can up-regulate the expression of invasion and migration factors (MMP2 and MMP9) and anti-apoptotic factor Bcl-2, and down-regulate the expression of apoptotic factor Bax, which ultimately promotes cell proliferation, invasion, and metastasis of HNSCC (19).

In gliomas, the transcription factor AP-4 can bind to the LINC00520 promoter region and activate the transcription of LINC00520, while miR-520f-3p can inhibit the expression of TFAP4, forming a TFAP4/LINC00520/miR-520f-3p/TFAP4 feedback loop, thereby regulating the malignant biological behavior of glioma cells (9).

Ubiquitin-specific protease 39 (USP39) plays a carcinogenic effect in various human tumors and can promote tumor cell growth, invasion, and migration (30). In NPC, LINC00520 is upregulated in NPC tissues and cells, and LINC00520 can sponge miR-26b-3p, thereby increasing the expression of USP39. NPC patients with high expression of LINC00520 have a poor prognosis. Knockdown of LINC00520 can inhibit the proliferation of NPC cells *in vitro* and tumor growth *in vivo*, and exert a tumor suppressor effect (21).

CCNE2 is a cell cycle-related protein that can promote tumor cell migration and invasion (31). In NSCLC, LINC00520 can promote the proliferation, migration, and invasion of NSCLC cells through the LINC00520/miR-577/CCNE2 axis, inhibit cell apoptosis, and promote the progress of NSCLC (10).

In summary, LINC00520 can participate in the growth and development of cancer cells through sponging miRNAs. Besides, the enhancement of the LINC0052/miR-27b-3p/OSMR axis can promote the progress of AKI (5).

## The LINC00520-Related Signaling Pathways

**Figure 2:**
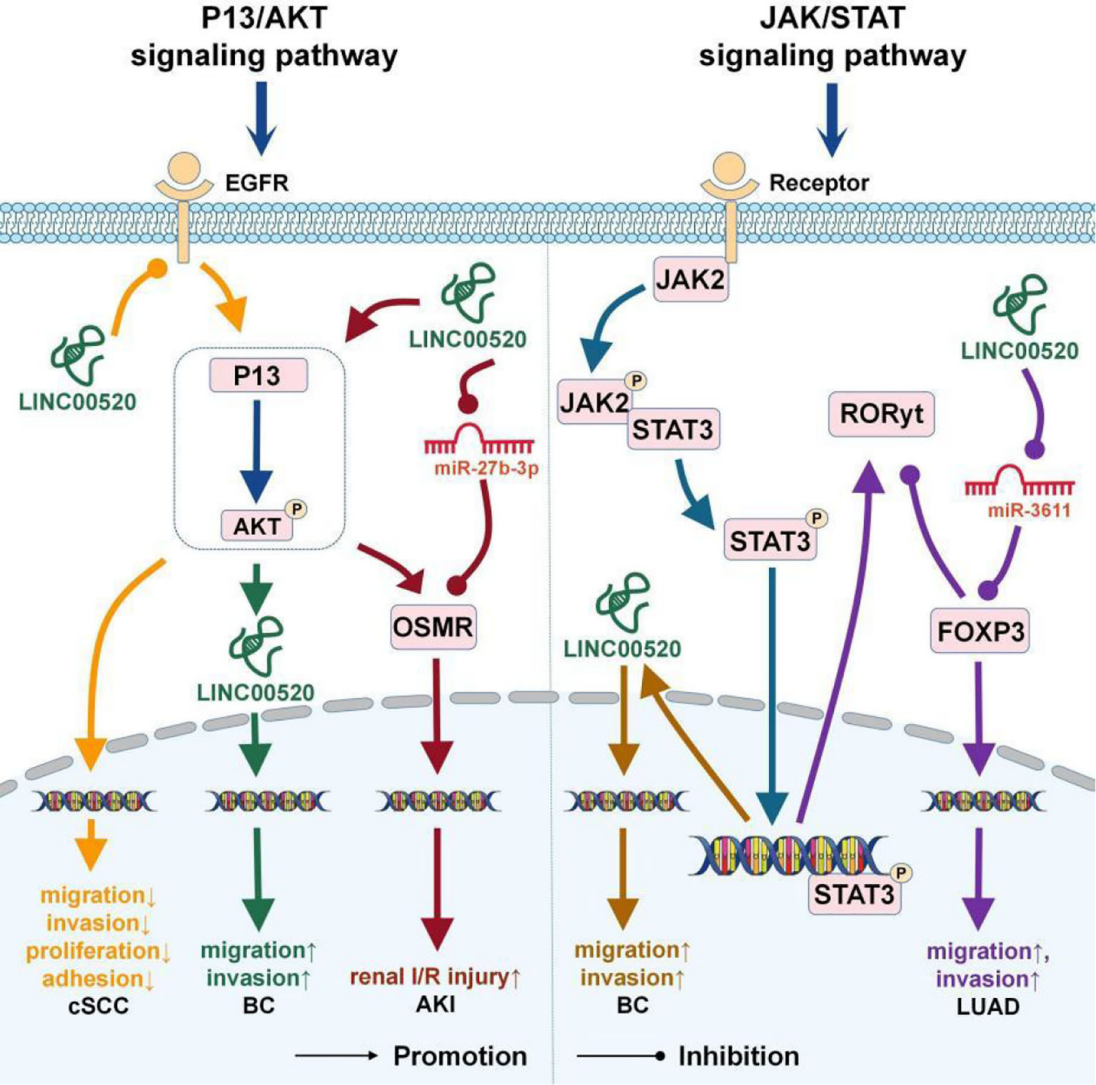
The role of LINC00520 in the P13/AKT and JAK/STAT signaling pathways. BC, breast cancer; cSCC, cutaneous squamous cell carcinoma; LUAD, lung adenocarcinoma; AKI, acute kidney injury.

LINC00520 participates in the regulation of the PI3K/AKT signaling pathway (5, 8, 22) and JAK/STAT signaling pathway (8, 13) (**Figure 2**).

### The PI3K/AKT Signaling Pathway

The PI3K/Akt signaling pathway is a very important intracellular signaling pathway during the cell cycle. It is related to cell quiescence, proliferation, cancer, and longevity (32).

In BC, excessive activation of the PI3K pathway is closely related to repeated somatic mutations in PIK3CA (33). Oncogenic PIK3CA can induce breast epithelial cells to transform into immortalized cells and can up-regulate the expression of LINC00520. In BC cells, depletion of PIK3CA can lead to down-regulation of LINC00520, indicating that LINC00520 is regulated downstream of oncogenic PI3K (8).

In AKI, LINC00520 as the ceRNA of miR-27b-3p can affect the expression of OSMR (5). Oncostatin M (OSM) is a secreted cytokine, and OSMR is the receptor of OSM. The acute phase reaction of the kidney may be mediated by the OSM/OSMR (34). In AKI, LINC00520 can upregulate OSMR expression by inhibiting miR-27b-3p or activating the PI3K/AKT signaling pathway, thereby aggravating AKI *in vitro* and *in vivo* (5).

Mutations in EGFR tyrosine kinase activate anti-apoptotic signaling pathways, such as PI3K/Akt, JAK-STAT, and ERK/MAPK (35). PI3K is a downstream signaling molecule of EGFR and an important factor that regulates the proliferation or invasion of HNSCC cells (36). In cSCC, LINC00520 inhibits the activation of the PI3K/Akt signaling pathway by targeting EGFR (22) and then inhibits the invasion and metastasis of cSCC (22).

### The JAK/STAT Signaling Pathway

Janus kinase (JAK) and activator of transcription (STATs) signaling pathways are highly conserved, and dysfunction of the JAK/STAT signaling pathway promotes the occurrence and metastasis of cancer (37).

In myeloproliferative tumors, JAK2 is often mutated, which leads to constitutive activation of JAK/STAT3 signaling. Overactivation of STAT3 signaling occurs in most human cancers and is also associated with poor prognosis (38). In BC, Src can phosphorylate STAT3, which can then bind to the promoter region of LINC00520 and promote the transcription of LINC00520 (8).

Overexpression of FOXP3 significantly induces cancer cell proliferation, migration, and invasion (27). The half-life of FOXP3 is very short and is mainly controlled by the JAK/STAT signal pathway (39). In LUAD cells, LINC00520 up-regulates the expression of FOXP3 through sponging miR-3611 and subsequently inhibits the expression of RORyt in the JAK/STAT signaling pathway (13).

## The Transcription Factors in the Regulation of LINC00520 Expression

**Figure 3:**
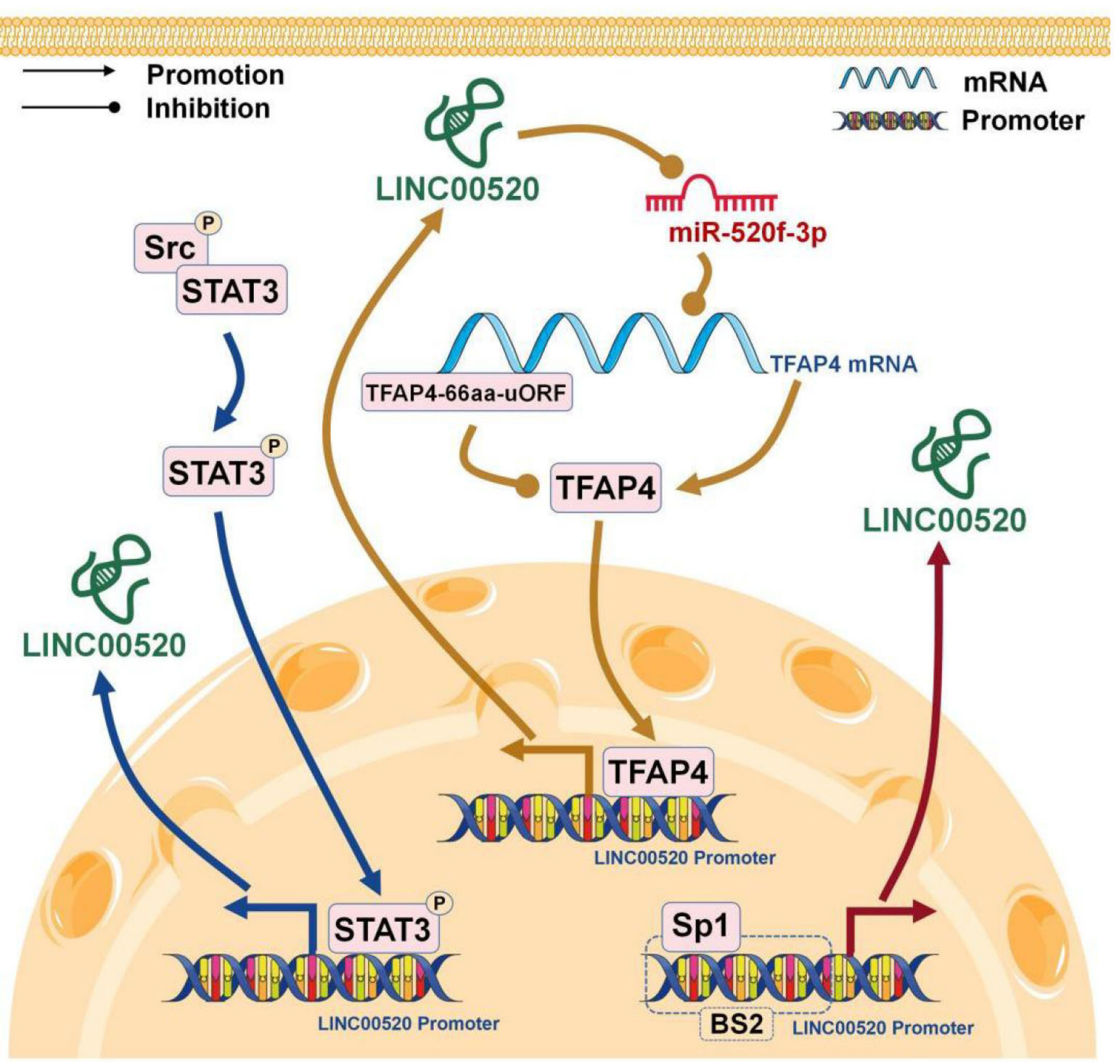
The transcription factors promotes the expression of LINC00520 in cancer.

The expression of lncRNA, like protein-coding gene, is related to the regulation of transcription factors (40). LIN00520 can be upregulated by SP1 in NSCLC (10), TFAP4 in glioma (9), and STAT3 in BC (8) (**Figure 3**).

Specific protein 1 (Sp1) comes from a member of the Sp/Kruppel-like transcription factor family, which also includes Sp2, Sp3, and Sp4. They are involved in a variety of basic biological processes and play carcinogenic effects in cell growth, differentiation, and apoptosis (41). The activation of SP1 is an important regulator in the progression of NSCLC (42). In NSCLC, SP1 directly binds to the promoter region of LINC00520, which further promotes the high expression of LINC00520 (10).

Transcription factor AP-4 (TFAP4) is a member of the basic helix-loop-helix-zipper family, which contains a basic DNA binding domain. TFAP4 can activate viral and cellular genes (43) by binding the CAGCTG DNA sequence. The upstream ORF (uORF) is the translation initiation element located at the 5’-untranslated region of eukaryotic mRNA. The main function of uORF is to regulate gene expression during gene translation (44). In gliomas, TFAP4-66aa-uORF inhibits the TFAP4/LINC00520/miR-520f-3p feedback loop by directly inhibiting TFAP4 expression, thereby attenuating glioma malignancies (9).

In the Src-transformed immortalized breast epithelial cells, Src phosphorylates STAT3 and phosphorylated STAT3 binds to the promoter region of LINC00520, thereby further promoting the expression of LINC00520 in BC, while depletion of STAT3 eliminates the up-regulation of LINC00520 induced by Src (8).

## LINC00520 in Cancers

**Figure 4:**
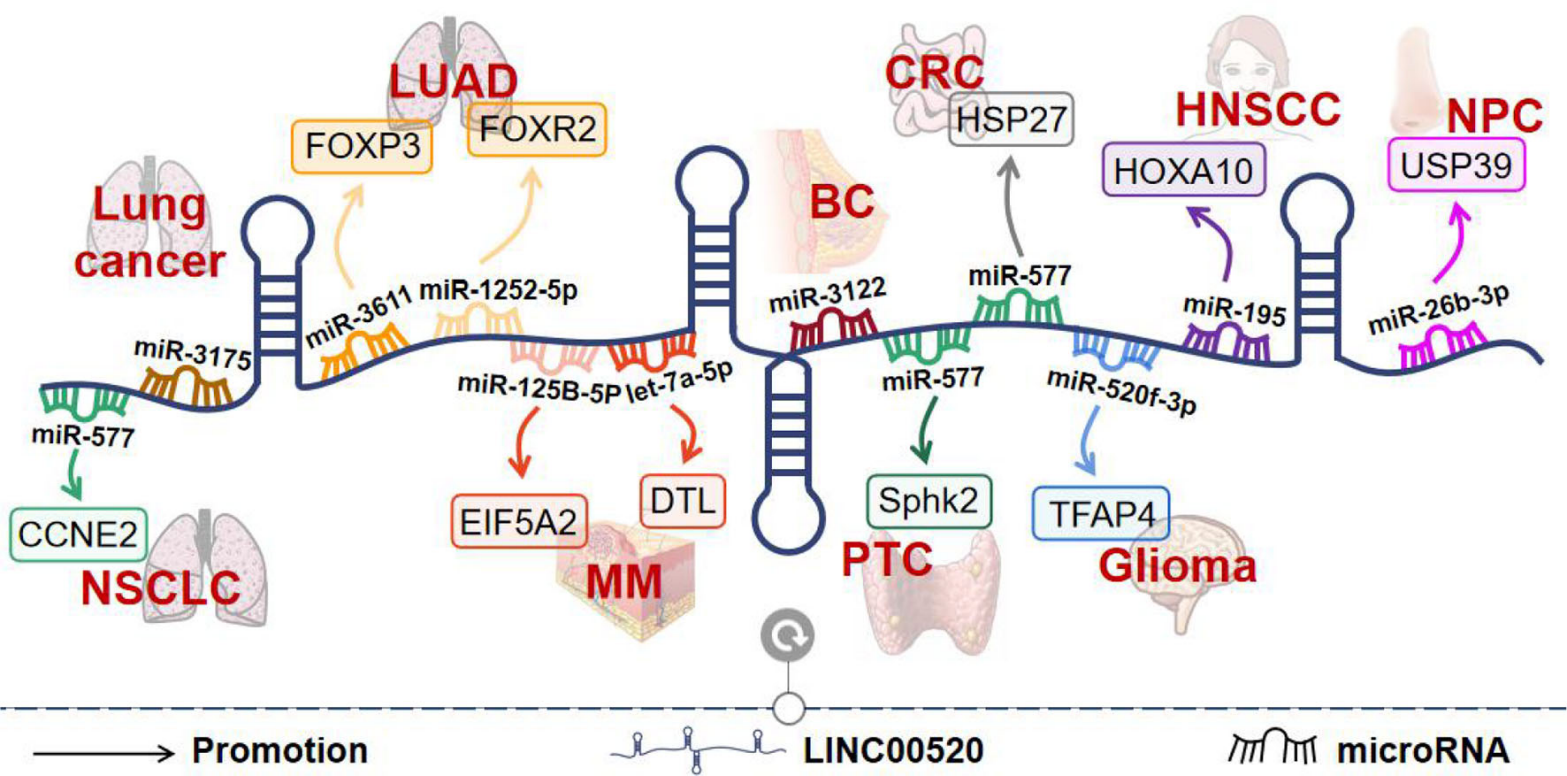
The molecular mechanisms of LINC00520 in different tumors. BC, breast cancer; PTC, papillary thyroid carcinoma; CRC, colorectal cancer; MM, melanoma; LUAD, lung adenocarcinoma; HNSCC, head and neck squamous cell carcinoma; NPC, nasopharyngeal carcinoma; NSCLC, non-small cell lung cancer.

In recent years, LINC00520 has been shown to play a key role in various cancers. These include reproductive system tumors (8, 4), respiratory system tumors (10–12), digestive system tumors (15), and nervous system tumors (9) (**Figure 4**).

### Reproductive System Tumors

BC is the most commonly diagnosed cancer in women and the second leading cause of cancer-related death in women (8). LINC00520 is upregulated in BC and promotes tumor invasion and metastasis (8). LINC00520 is upregulated in immortalized mammary epithelial cells transformed by oncogenic Src or oncogenic PI3K. Both loss-of-function and gain-of-function approaches have shown that LINC00520 promotes cell migration and cell invasion (8). In addition, a study has found an association between the LINC00520 gene polymorphism and BC susceptibility. The genetic variant of rs8012083 in LINC00520 was found to be a diagnostic biomarker for triple-negative BC. And rs12880540 may affect the expression of LINC00520 (4).

### Respiratory System Tumors

In China, the incidence and mortality of lung cancer rank first among malignant tumors, and the 5-year survival rate is only 19.7% (11). In the process of exploring the pathogenesis of lung cancer, the close relationship between non-coding RNAs including miRNAs and lncRNAs and tumors has been identified as a new research direction (11). LINC00520 is highly expressed in lung cancer tissues and cells. LINC00520 levels were consistently higher in stage III+IV patients than in stage I+II patients (11). In addition, patients with high LINC00520 expression had a shorter survival time. Knockdown of LINC00520 inhibited the proliferation, invasion, and migration of lung cancer cells. LINC00520 sponges miR-3175 to promote lung cancer progression (11).

NSCLC accounts for approximately 85% of lung cancers, and its incidence continues to rise worldwide (10). LINC00520 levels are elevated in NSCLC tissues and cells. Increased LINC00520 expression was significantly associated with advanced tumor stage and shorter survival time in NSCLC patients (10). Further functional studies found that downregulation of LINC00520 inhibited NSCLC cell proliferation, invasion, metastasis, and EMT, while promoted apoptosis (10). Mechanistically, LINC00520 was found to inhibit miR-577 expression as a ceRNA, thereby affecting the expression of CCNE2, a target gene of miR-577. Furthermore, SP1 can directly bind to the promoter region of LINC00520, thereby promoting its transcription (10).

Among NSCLC, LUAD is the most common histological subtype (12). The study found that LINC00520 was upregulated in LUAD. LINC00520 upregulation is associated with poor prognosis in patients with LUAD (13). Downregulation of LINC00520 inhibits LUAD cell proliferation and migration and induces apoptosis. Mechanistically, LINC00520 up-regulates FOXP3 expression by sponging miR-3611 in LUAD cells. In addition, FOXP3 was identified as a transcription factor that transcriptionally activates LINC00520 (13). Another study found that LINC00520 could directly bind to miR-1252-5p to regulate FOXR2 expression in LUAD tissues. miR-1252-5p reverses the functions of LINC00520 in LUAD cell progression *in vitro*, including proliferation, migration, and invasion (12). This suggests that LINC00520 may be an important biomarker and potential target in LUAD therapy.

### Digestive System Tumors

CRC is the third most common cancer worldwide, and understanding the occurrence of CRC is crucial for the design of molecular targeting (15). The study found that LINC00520 was highly expressed in CRC tissues and cell lines, and high expression of LINC00520 was associated with unfavorable clinicopathological parameters and shorter overall and disease-free survival in patients. Functionally, interference with LINC00520 resulted in a significant reduction in CRC cell proliferation, migration, colony-forming ability, and invasive ability. Mechanistically, LINC00520 acts as a ceRNA of miR-577, thereby increasing the expression of HSP27 (15).

### Nervous System Tumors

Gliomas are brain tumors originating from glial progenitor cells (9). The study found that LINC00520 was up-regulated in glioma malignancies. LINC00520 knockdown inhibited glioma cell proliferation, migration, and invasion, but promoted apoptosis. LINC00520 can promote the expression of TFAP4 by competitively binding to miR-520f-3p. In addition, TFAP4 activates the expression of LINC00520 by binding to the LINC00520 promoter region, forming a positive feedback loop of TFAP4/LINC00520/miR-520f-3p/TFAP4 (9).

## Conclusions

LINC00520 is highly expressed in 11 cancers, comprising lung cancer, NSCLC, LUAD, BC, PTC, CRC, MM, HNSCC, LSCC, glioma, and NPC. Based on clinical data, upregulation of LINC00520 is also considered to be an indicator of poor prognosis in lung cancer, NSCLC, LUAD, PTC, CRC, MM, HNSCC, LSCC, NPC, etc. Besides, upregulation of LINC00520 is also related to the reduced radiosensitivity of HNSCC.

LINC00520 is generally highly expressed in cancer, however, a study has shown that LINC00520 is down-regulated in cSCC. In cSCC, LINC00520 reduces the activity of the PI3K/AKT signaling pathway by inhibiting the expression of EGFR, thereby inhibiting the occurrence and development of cSCC. This study revealed that LINC00520 was down-regulated in cSCC through analyzing the data from GSE66359, and its cell experiments showed that overexpression of LINC00520 can inhibit EGFR, thereby hindering the development of cSCC. However, this study did not provide clinical samples to verify that LINC00520 was down-regulated in cSCC. In the future, more sophisticated experiments are needed to verify the function of LINC00520 in cSCC.

Current studies have shown that LINC00520 is mainly involved in the PI3K/AKT signaling pathway and the JAK/STAT signaling pathway. LINC00520 exhibits unique functions in various gene regulatory processes. LINC00520-miRNA-mRNA axis containing at least 10 miRNAs has been identified. The current study shows that the same lncRNA-miRNA axis may regulate different biological functions in different diseases, and the downstream miRNA of LINC00520 is miR-577 in NSCLC, CRC, and PTC. Second, the elements of the LINC00520-miRNA-mRNA axis can be affected by other molecules under stress conditions, making it difficult to fully explore the specific regulatory mechanism of LINC00520. Furthermore, it is unclear whether the dynamic regulation of the LINC00520-miRNA-mRNA axis can be considered a marker of drug treatment efficacy. In addition, transcription factors are also involved in the regulation of LINC00520, including SP1 in NSCLC, TFAP4 in glioma, and STAT3 in BC. The participation of transcription factors makes the transcription factor-LINC00520-miRNA-transcription factor form a positive feedback loop, which is involved in the occurrence and development of tumors.

LncRNAs are involved in multiple signaling pathways related to cancer development and are aberrantly expressed in specific tumors, so they can be used to develop new strategies for diagnosing and targeting specific cancer subtypes (45). However, lncRNA-based therapies still have the following challenges. First, the number of lncRNAs aberrantly expressed in various cancers is still relatively high, and it is crucial to identify the most important lncRNAs associated with specific cancer types/subtypes (45). Second, lncRNA-related research is currently at an early stage, and the structural and functional information of most lncRNAs remains uncharacterized. The exact mechanism by which lncRNA-associated ceRNA networks are involved in cancer progression remains largely unknown. Furthermore, unlike protein-coding genes, lncRNAs are poorly conserved across species. Therefore, the experimental results of animal models require detailed *in vitro* experimental studies before they can be applied to clinical research (45). To fully realize the potential of lncRNAs in cancer diagnosis and targeted therapy, more detailed lncRNA information is required in the future to determine their cellular functions and roles in cancer.

This article reviews the diagnostic and prognostic value of abnormally high expression of LINC00520 in cancer. Besides, this work demonstrates the ceRNA network centered on LINC00520 and discusses the role of LINC00520 in the PI3K/AKT signaling pathway and JAK/STAT signaling pathway. This work proposes that the establishment of the transcription factor-LINC00520-microRNAs-mRNA networks is expected to provide new ideas for tumor diagnosis, intervention, prognosis, and treatment.

## Author Contributions

QZ, FZ, and SD contributed to the conception, design and final approval of the submitted version. QZ, JS, YW, and WR collected and analyzed literature. QZ, FZ, and SD contributed to manuscript writing. All the authors conceived and gave the approval of the final manuscript.

## Funding

This study was supported by the Medical and Health Science and Technology Program of Zhejiang Province (No. 2020RC088).

## Conflict of Interest

The authors declare that the research was conducted in the absence of any commercial or financial relationships that could be construed as a potential conflict of interest.

## Publisher’s Note

All claims expressed in this article are solely those of the authors and do not necessarily represent those of their affiliated organizations, or those of the publisher, the editors and the reviewers. Any product that may be evaluated in this article, or claim that may be made by its manufacturer, is not guaranteed or endorsed by the publisher.
